# Transcriptomic analysis of monocytes from HIV-positive men on antiretroviral therapy reveals effects of tobacco smoking on interferon and stress response systems associated with depressive symptoms

**DOI:** 10.1186/s40246-019-0247-x

**Published:** 2019-11-28

**Authors:** David R. Lorenz, Vikas Misra, Dana Gabuzda

**Affiliations:** Department of Cancer Immunology and Virology, Dana-Farber Cancer Institute, Center for Life Science 1010, 450 Brookline Avenue, Boston, MA 02215 USA

**Keywords:** HIV, Tobacco, Smoking, Transcriptomics, Monocytes, Immune response, Interferon response, Stress response, Mitochondria, Depression

## Abstract

**Background:**

Tobacco smoking induces immunomodulatory and pro-inflammatory effects associated with transcriptome changes in monocytes and other immune cell types. While smoking is prevalent in HIV-infected (HIV+) individuals, few studies have investigated its effects on gene expression in this population. Here, we report whole-transcriptome analyses of 125 peripheral blood monocyte samples from ART-treated HIV+ and uninfected (HIV−) men enrolled in the Multicenter AIDS Cohort Study (MACS) (*n =* 25 HIV+ smokers, *n* = 60 HIV+ non-smokers, *n* = 40 HIV− non-smoking controls). Gene expression profiling was performed using Illumina HumanHT-12 Expression BeadChip microarrays. Differential expression analysis was performed with weighted linear regression models using the R *limma* package, followed by functional enrichment and Ingenuity Pathway analyses.

**Results:**

A total of 286 genes were differentially expressed in monocytes from HIV+ smokers compared with HIV− non-smokers; upregulated genes (*n* = 180) were enriched for immune and interferon response, chemical/stress response, mitochondria, and extracellular vesicle gene ontology (GO) terms. Expression of genes related to immune/interferon responses (*AIM2*, *FCGR1A-B*, *IFI16*, *SP100*), stress/chemical responses (*APAF1*, *HSPD1*, *KLF4*), and mitochondrial function (*CISD1*, *MTHFD2*, *SQOR*) was upregulated in HIV+ non-smokers and further increased in HIV+ smokers. Gene expression changes associated with smoking in previous studies of human monocytes were also observed (*SASH1*, *STAB1*, *PID1*, *MMP25*). Depressive symptoms (CES-D scores ≥ 16) were more prevalent in HIV+ tobacco smokers compared with HIV+ and HIV− non-smokers (50% vs. 26% and 13%, respectively; *p* = 0.007), and upregulation of immune/interferon response genes, including *IFI35*, *IFNAR1*, *OAS1-2*, *STAT1*, and *SP100*, was associated with depressive symptoms in logistic regression models adjusted for HIV status and smoking (*p* < 0.05). Network models linked the Stat1-mediated interferon pathway to transcriptional regulator Klf4 and smoking-associated toll-like receptor scaffolding protein Sash1, suggesting inter-relationships between smoking-associated genes, control of monocyte differentiation, and interferon-mediated inflammatory responses.

**Conclusions:**

This study characterizes immune, interferon, stress response, and mitochondrial-associated gene expression changes in monocytes from HIV+ tobacco smokers, and identifies augmented interferon and stress responses associated with depressive symptoms. These findings help to explain complex interrelationships between pro-inflammatory effects of HIV and smoking, and their combined impact on comorbidities prevalent in HIV+ individuals.

## Background

Tobacco smoking is a major risk factor for lung and cardiovascular diseases, and remains a significant cause of mortality and morbidity in the USA and globally [1, 2]. Among persons living with human immunodeficiency virus 1 (HIV+), the prevalence of tobacco smoking remains high despite recent declines in its prevalence in other populations [3]. Estimates of the proportion of current smokers among HIV+ adults in the USA range from 33–42%, approximately twice as high as in the general population, and HIV+ individuals are less likely to quit smoking [3, 4]. Furthermore, HIV+ individuals have higher rates of comorbidities associated with smoking compared with persons in the general population, including lung cancer [5], infectious and chronic obstructive pulmonary diseases (COPD) [6, 7], and cardiovascular disease [8–10].

Smoke from tobacco combustion contains over 1000 chemicals including carcinogens, toxins, particulates, and reactive oxidative species (ROS) [11, 12]. Cellular responses to these insults are mediated in part through immune cell activation and modulation of inflammation, oxidative stress, and DNA damage responses [11–13]. While smoking-associated signaling pathways and their effects on cellular transcriptional programs have been previously described in immune cells [11], the molecular mechanisms underlying responses to tobacco smoking, especially in HIV+ tobacco smokers, remain poorly understood.

Previous studies in the general population have characterized transcriptomic profiles of immune cell responses in peripheral blood from smokers, including monocytes [14–16], peripheral blood mononuclear cells (PBMCs) [17, 18], and other cell types [19–22]. These studies identified gene expression signatures that distinguish current, former, and never smokers [20–22], and COPD-specific gene expression profiles shared in peripheral blood monocytes and alveolar macrophages [23]. Monocytes are innate immune cells involved in inflammatory responses activated by pathogens and other external agents including tobacco smoke [14], are associated with plaque formation in atherosclerotic cardiovascular disease [24], and contribute to increased risk of atherosclerosis in HIV+ individuals on antiretroviral therapy (ART) [25]. Previous transcriptome-wide studies of monocytes from tobacco smokers identified new genes associated with smoking and atherosclerosis in the general population [15, 26], yet similar studies in HIV+ individuals are limited. We hypothesized that the combination of HIV infection and tobacco smoking would have additive effects on stress and inflammatory response gene expression profiles in monocytes. The aims of this study were to investigate the effect of tobacco smoking on transcriptome-wide gene expression changes in peripheral blood monocytes from HIV+ men enrolled in the Multicenter AIDS Cohort study (MACS), an ongoing prospective study of HIV+ and uninfected (HIV-) individuals with similar lifestyle and demographic characteristics.

## Results

### Study cohort characteristics

**Fig. 1:**
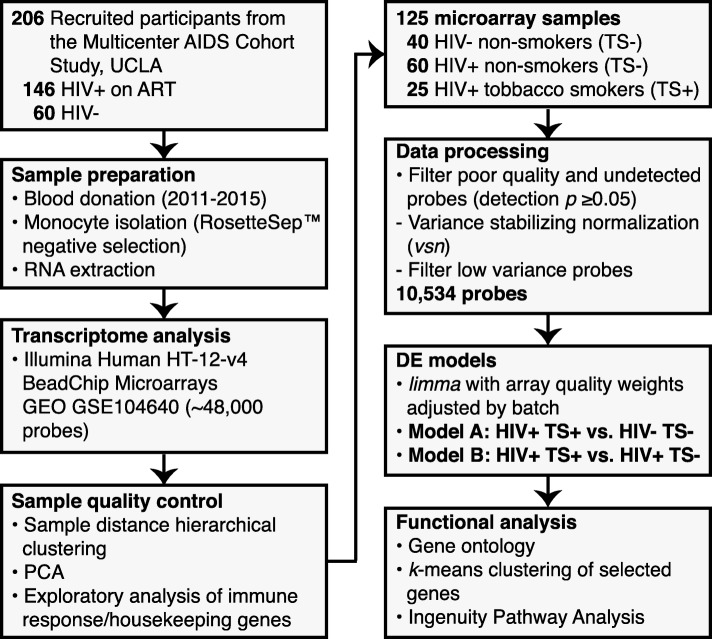
Study workflow diagram. Overview of sample processing and bioinformatic analysis steps with numbers of included participants, samples, and microarray probes. DE, differential expression; GEO, NCBI Gene Expression Omnibus, PCA, principal component analysis; TS+, tobacco smoker; TS−, non-smoker

Characteristics of the study population are shown in Table 1 and an overview of the cohort selection is shown in Fig. 1. The cohort was predominantly white and middle-aged (median 54 years), with ≥ 12 years of education (89%). HIV+ participants were younger (median age [IQR]: 49 [40-55], 53 [45–61], and 59 [54–65] years for HIV+ smokers (TS+), HIV+ non-smokers (TS−), and HIV− TS−, respectively; *p* < 0.001), with more persons of black, Hispanic, or other race/ethnicity among HIV+ compared with HIV− participants (*p* = 0.001). The proportion of former smokers was similar between HIV− and HIV+ non-smokers (65% vs. 68%). Ten HIV+ TS+ and ten HIV+ TS− participants also reported daily or weekly marijuana use during the prior six months. Depressive symptoms (CES-D score ≥ 16) were more prevalent among HIV+ TS+ and TS− compared with HIV− TS− participants (50% and 27%, vs. 13%, respectively; *p* = 0.007), while there was no differences in prevalence of alcohol use, cocaine use, BMI, and HCV serostatus. HIV disease markers including CD4+ T cell counts (611 vs. 598 cells/μl; *p* = 0.231) and HIV viral loads (85% vs. 91% with < 200 copies/ml; *p* = 0.701) were similar between smoking and non-smoking HIV+ participants.

**Table 1:** Demographic and clinical characteristics of the study cohort

*n*	HIV−non-smokers	HIV+non-smokers	HIV+tobacco smokers	*p*
	40	60	25	
Age (median [IQR])	58.5 [54.0, 64.0]	53.0 [45.8, 61.0]	49.0 [40.0, 55.0]	< 0.001
Race				0.001
White	30 (75.0)	32 (53.3)	6 (24.0)	
Black	3 (7.5)	9 (15.0)	9 (36.0)	
Hispanic/Other	7 (17.5)	19 (31.7)	10 (40.0)	
Education ≤ 12 years	1 (2.5)	8 (13.3)	6 (24.0)	0.021
Tobacco smoking				< 0.001
Current	0 (0.0)	0 (0.0)	25 (100.0)	
Former	26 (65.0)	41 (68.3)	0 (0.0)	
Never	14 (35.0)	19 (31.7)	0 (0.0)	
Marijuana smoking^a^	0 (0.0)	10 (16.7)	10 (40.0)	< 0.001
Alcohol use^a^	13 (32.5)	18 (30.0)	10 (40.0)	0.657
Cocaine use^a^	1 (2.5)	1 (1.7)	0 (0.0)	0.999
CES-D score				0.007
0– < 16	33 (86.8)	44 (73.3)	12 (50.0)	
≥ 16	5 (13.2)	16 (26.7)	12 (50.0)	
BMI (kg/m^2^, median [IQR])	26.1 [22.9, 30.5]	25.2 [22.9, 27.8]	26.9 [22.3, 29.3]	0.630
HCV positive	0 (0.0)	1 (1.7)	1 (5.0)	0.368
HIV viral load (copies/ml, median [IQR])	––	10.0 [10.0, 10.0]	10.0 [10.0, 65.5]	0.093
HIV viral load ≤200 copies/ml	––	53 (91.4)	17 (85.0)	0.416
CD4 count (cells/μl, median [IQR])	915 [750, 1235]	598 [478, 854]	611 [318, 758]	< 0.001

All data are *n* (%) unless otherwise indicated; *p* value denotes Fisher’s exact test, or Kruskal-Wallis rank sum test for continuous variables

^a^Daily or weekly marijuana smoking, alcohol use, or cocaine use

### Identification and functional characterization of smoking-associated genes

**Fig. 2:**
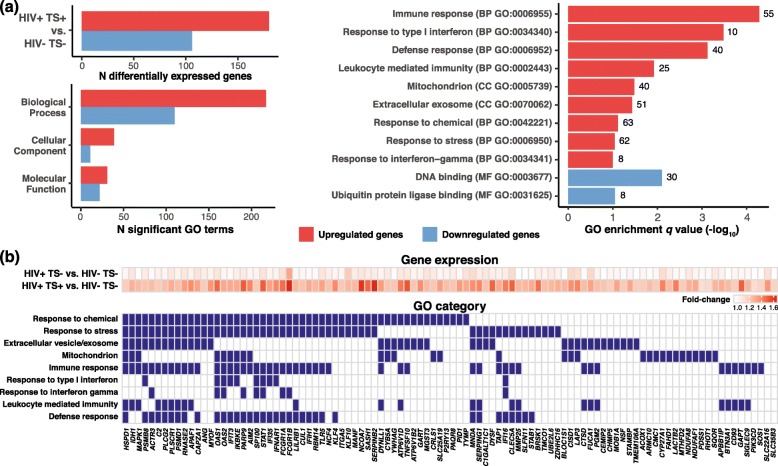
Overview and functional categories of differentially expressed genes in peripheral blood monocytes from HIV+ tobacco smokers vs. HIV- non-smokers. **a** Number of differentially expressed genes (FDR-adjusted *p* value < 0.15, absolute log_2_ FC > 0.25) (top left panel), number of enriched GO terms (*p* < 0.05) by ontology and direction of expression change (bottom left panel), and representative enriched GO terms for sets of up- and down-regulated genes (FDR-adjusted *p* value < 0.10; right panel) from models contrasting HIV+ smokers vs. non-smokers. Full expression and GO enrichment results are included in Additional file 2: Tables S1 and S2. **b** Average fold-change differences in HIV+ smokers and non-smokers compared with uninfected individuals (top panel) and associated GO categories for 100 selected upregulated genes mapping to significantly enriched GO terms (bottom panel). Genes with no mapped GO terms but known associations with tobacco smoking in monocytes reported in the literature (*SLC2A16*, *SLC35B3*) were included. GO, Gene Ontology; FDR, false discovery rate; TS+, tobacco smoker; TS−, non-smoker

An overview of the data processing pipeline is shown in Fig. 1, and a summary of differentially expressed (DE) genes distinguishing HIV+ TS+ vs. HIV− TS− samples and representative top enriched gene ontology (GO) categories are shown in Fig. 2a. Two hundred nine upregulated probes mapping to 180 genes and 116 downregulated probes mapping to 106 genes were identified as DE and included in GO enrichment analysis (absolute log_2_ fold-change (FC) > 0.25, FDR-adjusted *p* value < 0.15). Most significantly enriched GO terms for upregulated genes mapped to the Biological Process (BP) ontology, with enriched terms related to immune responses, responses to interferon, responses to chemicals and stress, defense response, and leukocyte mediated immunity. Enriched Cellular Component (CC) ontology terms for upregulated genes included mitochondrion and extracellular vesicles/exosome. In contrast, there were fewer enriched terms for differentially expressed downregulated genes, which mapped to DNA binding and ubiquitin protein ligase binding. Subsequent analyses therefore focused on upregulated genes. The full set of fold-changes and enriched GO terms are included in Additional file 2: Tables S1 and S2. Models comparing HIV+ TS+ vs. TS− samples yielded similar but smaller gene sets compared with models comparing HIV+ TS+ vs. HIV− TS− samples (Additional file 1: Figure S1a; Additional file 2: Tables S3 and S4). Additional models and GO analyses comparing HIV+ TS− vs. HIV− TS− samples revealed few DE genes and no significantly enriched GO terms (Additional file 2: Tables S5 and S6). In a separate sensitivity analysis excluding six HIV+ participants with HIV viral load > 200 copies/ml, differentially expressed gene sets overlapped closely with those from models including all participants (Additional file 1: Figure S1b–S1d; Additional file 2: Tables S7–S10), indicating these results were not driven by participants with unsuppressed viral load.

A representative subset of 100 differentially expressed genes upregulated in HIV+ TS+ vs. HIV− TS− samples associated with enriched GO terms are shown in Fig. 2b. This included canonical genes for activated immune/interferon signaling pathways (*STAT1*, *OAS1-2*, *FCGR1A-B, IFI6, IFIH1*, *IFIT3, IFI35*), and previously well-characterized genes induced by cellular/chemical stress (*HSPD1*, *IDH1*, *APAF1*, *SERPINB2*). Most upregulated genes mapped to multiple GO terms related to immune/interferon responses, cellular/chemical stress responses, and defense responses including response to viruses, or to GO cellular component terms for extracellular vesicles/exosomes or mitochondria. This set also included genes associated with smoking in previous studies of peripheral blood monocytes in the general population (*SASH1*, *FUCA1*, *PID1*, *STAB1*, *MMP25*) [14–16], PBMCs (*SASH1*, *FUCA1*, *PID1*, *SERPINB2*) [17, 18], or other blood cell types (*SASH1*, *FUCA1*, *SERPINB2*, *SERPING1*, *SLC22A16*, *SLC35B3*) [20, 21].

### Functional characterization of gene sets correlated with tobacco smoking and depressive symptoms

**Fig. 3:**
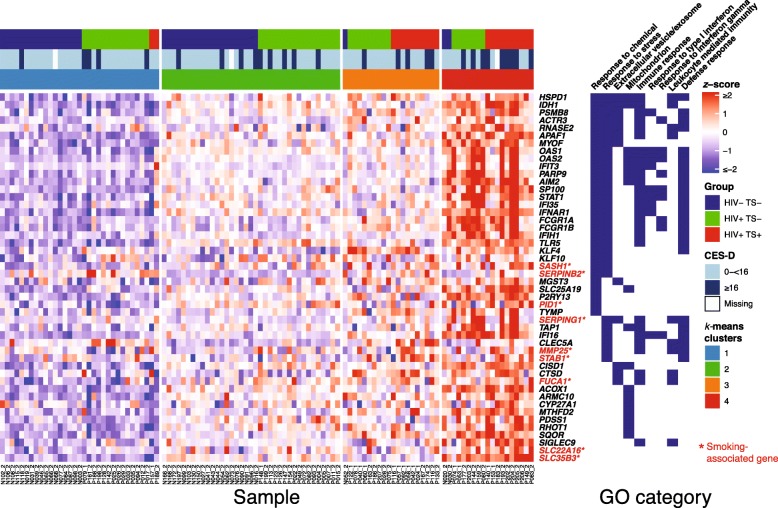
Gene expression heatmap and GO ontology of selected smoking-associated genes by HIV status and tobacco smoking. Normalized expression values from 48 selected upregulated DE genes mapping to enriched GO terms (Fig. 2) were *z-*scored, samples were grouped by *k*-means clusters (optimal *k* = 4) and ordered by cluster, HIV status, and smoking. Colored bars above heatmap denote HIV and smoking groups and CES-D depression scores (≥ 16 indicates risk for clinical depression [55]). Genes associated with smoking in previous studies of monocytes in the general population marked in red with asterisks. CES-D, Center for Epidemiological Studies Depression Scale; GO, Gene Ontology; TS+, tobacco smoker; TS−, non-smoker

Next, we assessed associations between clinical characteristics and expression of selected genes by clustering and further probe-level analyses. Given the high prevalence of depressive symptoms in HIV+ tobacco smokers (Table 1), and previous studies reporting an association between depressive symptoms and increased interferon and inflammatory gene expression in immune cells [27–29], we further evaluated smoking-associated gene expression in participants with depressive symptoms (CES-D score ≥ 16). *K*-means clustering was performed for *z*-scored, normalized probe intensities of 48 DE genes (log_2_ fold-change > 0.25, FDR-adjusted *p* value < 0.10) representing key GO functional categories, including genes associated with smoking in previous studies of peripheral blood monocytes and other blood cell types (Fig. 3). While this analysis revealed expression heterogeneity between individuals in HIV and smoking exposure groups, *k*-means clustering identified four sample clusters, in which HIV+ TS+ samples comprised the largest subset in clusters three and four. Participants with CES-D scores ≥ 16 indicating high depressive symptoms clustered with HIV+ TS+ samples and exhibited relatively high expression of genes associated with immune/interferon responses (*OAS1-2*, *FCGR1A-B*, *IFI16*, *IFIH1*, *IFIT3*, *IFI35*), while other participant demographic and clinical characteristics showed no clear association with this profile (data not shown).

To further assess the association between immune/interferon response gene expression and depressive symptoms, multivariate logistic regression models adjusted by HIV status and smoking were fit for each gene mapping to GO terms for immune response, response to type I interferon, or response to interferon-γ (Fig. 2). Increased expression of eight genes (*IFI35*, *IFNAR1*, *OAS1*, *OAS2*, *PGM2*, *PSMD1*, *SP100*, *STAT1*) was associated with CES-D score ≥ 16 modeled as either a continuous (per one glog_2_, i.e*.*, 2-fold increase) or categorical variable (highest vs. middle and lowest expression tertile; Table 2). Consistent with these models, expression of these genes was increased in HIV+ TS+ samples from participants with CES-D score ≥ 16 compared with samples from non-depressed HIV− TS− participants (*p <* 0.05, Wilcoxon rank-sum test; Additional file 1: Figure S2).

**Table 2:** Immune/interferon response genes associated with CES-D depression scores ≥ 16 in logistic regression models adjusted for HIV status and tobacco smoking

Gene	Description	Continuous expression (per glog_2_ increase)		Categorical expression (highest vs. middle and lowest tertiles)	
		OR (95% CI)	*p*	OR (95% CI)	*p*
*IFI35*	Interferon induced protein 35	2.49 (1.06, 6.29)	0.041	1.93 (0.78, 4.76)	0.15
*IFNAR1*	Interferon alpha and beta receptor subunit 1	3.07 (1.00, 10.45)	0.059	2.72 (1.09, 6.85)	0.032
*OAS1*	2'-5'-oligoadenylate synthetase 1	1.97 (1.07, 3.85)	0.035	2.35 (0.95, 5.87)	0.064
*OAS2*	2'-5'-oligoadenylate synthetase 2	2.37 (1.07, 5.68)	0.039	2.67 (1.07, 6.72)	0.035
*PGM2*	Phosphoglucomutase 2	9.35 (1.63, 64.55)	0.016	3.50 (1.42, 8.89)	0.0070
*PSMD1*	Proteasome 26S subunit, non-ATPase 1	16.80 (2.33, 157.17)	0.0081	4.14 (1.66, 10.71)	0.0026
*STAT1*	Signal transducer and activator of transcription 1	1.99 (0.92, 4.46)	0.083	2.41 (0.97, 6.01)	0.056
*SP100*	SP100 nuclear antigen	6.77 (0.96, 52.49)	0.058	1.96 (0.76, 5.00)	0.16

Separate multivariable logistic regression models were fit for each indicated gene with CES-D depression scores ≥ 16 (*n =* 30) vs. < 16 as outcome variable for *n =* 115 samples with available CES-D data. All models were adjusted by a categorical variable for HIV and smoking status. *CES-D*, Centers for Epidemiologic Studies Depression; *CI*, confidence interval; *OR*, odds ratio

**Fig. 4:**
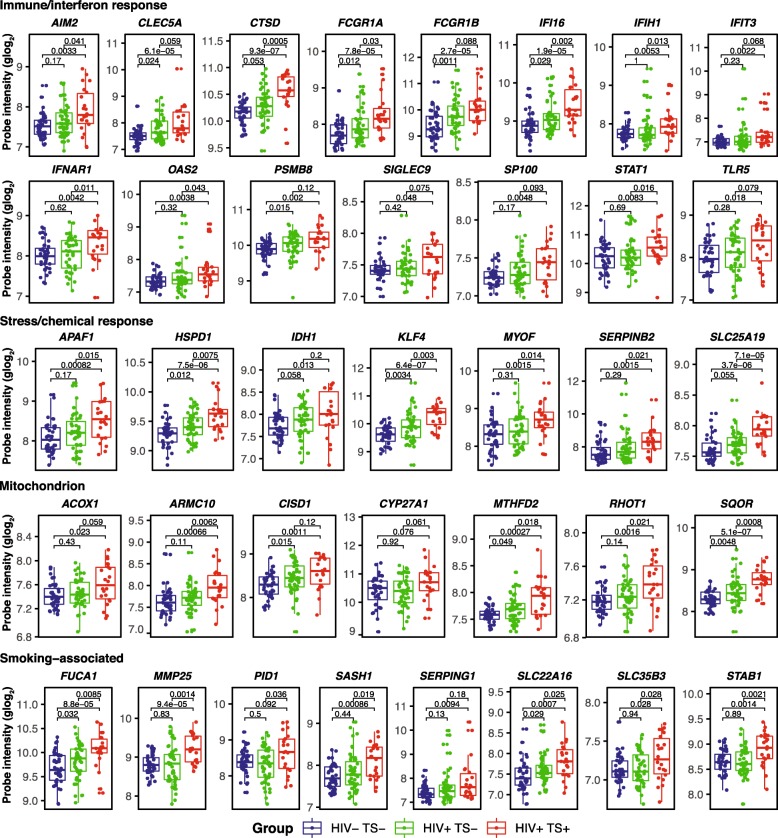
Expression levels of selected immune/interferon response, stress/chemical response, mitochondrion, and smoking-associated genes by HIV status and smoking. Boxplots of normalized gene expression levels for selected genes upregulated in HIV+ smokers compared with HIV− non-smokers. Horizontal bars denote medians, boxes span IQRs, whiskers extend to 1.5 × IQR. Dots denote expression values from individual arrays. Probe intensities were normalized and generalized-log_2_ transformed using the R/Bioconductor *vsn* package. Groups were compared using the Wilcoxon rank-sum test. IQR, interquartile range; TS+, tobacco smoker; TS−, non-smoker

To further evaluate and compare monocyte gene expression from HIV+ TS+ and TS− vs. HIV− TS− samples, probe intensities of selected immune/interferon response, stress/chemical response, mitochondrion-associated, and smoking-associated genes were examined with Wilcoxon rank-sum tests (Fig. 4). Most selected genes displayed increased expression in HIV+ vs. HIV− TS− samples and an additive increase in HIV+ TS+ samples, despite expression heterogeneity within groups. Genes displaying this trend with significant increases in all comparisons (*p* < 0.05, Wilcoxon rank-sum test) or near-significant increases included known key immune/interferon response genes (*AIM2*, *CLEC5A*, *CTSD, FCGR1A*, *FCGR1B*, *IFI16*, *PSMB8*, *SP100*), stress/chemical response-associated heat-shock chaperonin protein *HSPD1*, Krüppel-like factor transcriptional regulator *KLF4*, and mitochondrion-associated genes *CISD1* (CDGSH iron sulfur domain redox-active protein) and *MTHFD2* (methylenetetrahydrofolate dehydrogenase). Smoking-associated genes identified in previous studies of peripheral blood monocytes (*SASH1*, *PID1*, *STAB1*, *MMP25*) were positively associated with smoking but not HIV status. Stratified comparisons were repeated including samples from ten HIV+ TS− marijuana users, which were excluded from previous analyses (Additional file 1: Figure S3). A small proportion of these genes were upregulated (*ARMC10*, *IFIH1*, *IFIT3*, *MMP25*, *SERPING1*, *STAT1*; *p* < 0.10) and none was downregulated in samples from HIV+ marijuana users compared with HIV− and HIV+ TS− non-users, and effect sizes were weaker than comparisons between TS+ and TS− samples in Fig. 4.

### Network analysis of *SASH1*, Stat1/interferon signaling, and stress response pathways

**Fig. 5:**
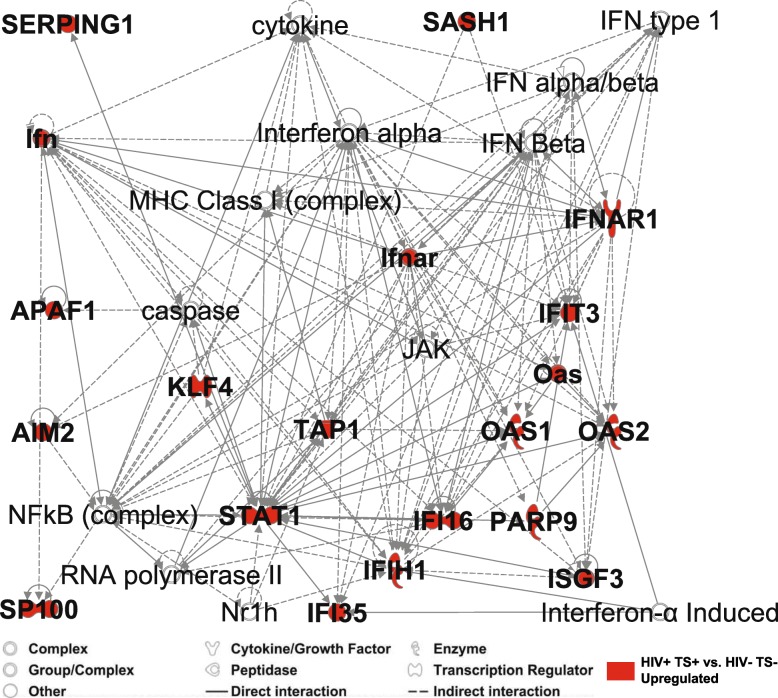
Tobacco-smoking associated genes map to interconnected interferon and stress response signaling networks. Ingenuity Pathway Analysis network diagram connecting Sash1 with selected interferon signaling molecules and stress response transcription factors. Sash1 encodes a scaffolding protein in the toll-like receptor (TLR4) pathway and is upregulated in tobacco smokers in the present and previous studies [14–18, 20]. The Krüppel-like transcription factor Klf4 associated with smoking in other cell types [50, 51] was also included. Nodes highlighted in red were upregulated in models contrasting HIV+ smokers vs. HIV− non-smokers (FDR-adjusted *p* value < 0.15, absolute log_2_ FC > 0.25); edges denote previously described positive or negative regulatory interaction and associations

*SASH1* encodes a SAM and SH3 domain containing scaffold protein involved in toll-like receptor 4 (TLR4) mediated signaling, which induces the NF-ΚB pathway and inflammatory cytokine production when activated [26, 30, 31]. *SASH1* was among the top ten DE genes (Additional file 2: Tables S1 and S3), and previous studies reported increased *SASH1* expression in monocytes and other blood cells from smokers vs. non-smokers [14–17, 20, 21, 26]. However, studies investigating the functional significance of this association are limited [26]. Network analysis using Ingenuity Pathway Analysis software and database (IPA) was performed to connect *SASH1* to immune and interferon response genes upregulated in samples from HIV+ smokers (Fig. 5). This analysis identified a high-scoring network connecting *SASH1* to interferon-induced gene *IFIT3* and transcriptional regulator *STAT1*, which in turn activates other key transcriptional regulators (NF-ΚB, interferon-induced *IFI16*, and *KLF4*, involved in monocyte differentiation). Pearson’s correlation analyses comparing *SASH1* expression with the set of 10,534 expressed probes showed significant relationships between *SASH1* and network genes *SP100* (SP100 nuclear antigen, induced by interferon; *r =* 0.454, FDR-adjusted *p* = 0.0032), and *APAF1* (apoptotic peptidase activating factor; *r* = 0.538, FDR-adjusted *p* = 0.001), as well as mitochondria-associated genes (*ARMC10*, *CISD1*, *P2RY13*, *RHOT1*), immune/stress response pathway genes (*IDH1*, *TLR5*), and tobacco smoking-associated genes (*FUCA1*, *PID1*) (Additional file 1: Figure S4). These results are consistent with a model in which Sash1 functions upstream of Stat1, which then induces expression of transcriptional regulators Klf4 and Ifi16 and associated network of interferon-associated, stress response, and nuclear-encoded mitochondrial oxidation genes.

## Discussion

To our knowledge, this is the first transcriptomic study investigating the effects of tobacco smoking on monocyte gene expression in ART-treated HIV-infected individuals. Samples were collected between 2011 and 2015, and regular cigarettes (not e-cigarettes) were the most likely route of exposure. A total of 180 upregulated and 106 down-regulated genes were identified in adjusted models contrasting HIV+ smokers vs. HIV− non-smokers. As reported previously in transcriptomic studies of monocytes from the general population, DE genes changed < 2-fold in smokers vs. non-smokers [14–16]. In contrast to previous studies of monocytes from ART-naïve or mostly viremic HIV+ participants [32, 33], few genes were differentially expressed between ART-treated HIV+ vs. HIV− non-smokers (Fig. 2 and data not shown), consistent with a previous study of gene expression changes in HIV+ persons on ART with controlled viral loads [34]. Genes positively associated with tobacco smoking were enriched with GO terms for stress/chemical response, immune/interferon response, leukocyte mediated immunity, defense responses including response to virus, and for cellular component terms mapping to mitochondria and extracellular vesicles/exosome. Compared with HIV− non-smokers, DE genes in functionally relevant categories displayed increased expression in HIV+ non-smokers, and further additive increase in HIV+ smokers, including genes related to immune activation and inflammation via interferon signaling (*AIM2*, *CLEC5A*, *CTSD*, *FCGR1A*, *FCGR1B*, *IFI16*, *PSMB8*, *SP100*), stress response (*HSPD1*, *KLF4*), and mitochondrial localization (*CISD1*, *MTHFD2*, *SQOR*). These results suggest that tobacco smoking augments the effects of HIV infection on immune-response, inflammation, and stress-response gene expression in monocytes. Further studies are needed to determine the association between induction of these transcriptional programs and inflammation-related comorbidities such as cardiovascular disease in HIV-infected individuals.

Responses to tobacco smoke exposure involve a variety of mechanisms influenced by host genetic susceptibility, lifestyle factors, and exposure history [12]. Direct activation of airway epithelial and innate immune cells, particularly by ROS, involves stimulation of pro-inflammatory signaling pathways, followed by induced expression and secretion of inflammatory cytokines (*e.g.*, tumor necrosis factor (TNF) α and IL-8) promoting activation and recruitment of additional immune cells including T-cells, macrophages, neutrophils, and dendritic cells [11, 13]. Enriched gene set functional categories identified here are consistent with this mechanism, and overlap with gene sets reported in previous studies of smoking in human monocytes [15, 16, 20, 22, 35]. The enrichment of GO terms for extracellular vesicle/exosome localization was unexpected given that few studies have investigated their association with smoking. In light of a recent proteomics study identifying markers of oxidative stress in exosomes derived from HIV-infected subjects on ART [36], and an additional study reporting an effect of cigarette smoke extract on exosome cargo and HIV replication in monocyte-derived cell lines [37], further study is warranted to characterize the effects of smoking on exosomes in HIV+ individuals.

The proportion of HIV-infected individuals with CES-D score ≥ 16 indicating depressive symptoms was markedly higher in HIV+ smokers compared with HIV+ and HIV− non-smokers (50%, 27%, and 13%, respectively; Table 1), consistent with a previous study reporting higher prevalence of smoking among HIV+ persons with major depressive disorder [4]. An association between inflammation and depressive symptoms is well-established, though previous studies reported high inter-individual variance of inflammatory markers among depressed persons [28, 38]. Increased expression of canonical immune/interferon response induced genes, including *IFI35*, *IFNAR1*, *OAS1*, *OAS2*, *PGM2*, *PSMD1*, *SP100*, and *STAT1* was associated with depressive symptoms in logistic regression models adjusted for HIV status and smoking. These findings are consistent with previous studies reporting increased expression of immune activation, type I interferon response, and inflammation-associated genes in blood cells from persons with depression or stress disorders in the general population [38–40]. Increased prevalence of major depressive disorders has been reported in approximately one-quarter of HCV patients receiving interferon-alpha therapy, which is associated with induced immune activation and increased *STAT1* expression and signaling [41, 42]. Unexpectedly, there were only marginal associations between expression of immune/interferon response genes and CES-D score ≥ 16 in models adjusted by HIV status among non-smokers (data not shown), which may reflect the substantial variation in gene expression among HIV+ and HIV− non-smoking participants revealed by clustering analysis (Fig. 3), or biological subtypes of depression mediated via distinct pathways [29, 43]. Studies of larger cohorts are needed to better characterize mechanisms underlying the high prevalence depressive disorders in HIV-infected persons.

Genes associated with tobacco smoking in previous studies of human monocytes were upregulated in HIV+ smokers in the present study, including *MMP25*, *PID1*, *SLC22A16*, *SLC35B3*, and notably *SASH1*, *FUCA1*, *STAB1*, *SERPING1*, which were among 11 genes in a signature predictive for tobacco smoking in whole blood samples [21]. While consistently detected in monocytes or blood cells from smokers in previous studies [14–18, 20], the functional mechanisms underlying association of these genes with tobacco smoke exposure remain unclear, though one recent study identified increased *SASH1* in atherosclerotic carotid arteries of smokers [26]. Ten HIV+ subjects in our study reported daily or weekly marijuana use (most likely by smoke inhalation) and did not smoke tobacco; we therefore assessed expression of tobacco smoking-associated and other selected genes in these participants (Additional file 1: Figure S3). Six genes were upregulated and none down-regulated in HIV+ marijuana users compared with HIV+ non-smoking non-users (*ARMC10*, *IFIH1*, *IFIT3*, *MMP25*, *SERPING1*, *STAT1*; *p* < 0.10); thus, we found no obvious similarity between gene expression patterns associated with tobacco vs. marijuana use, though these results should be interpreted with caution given small sample sizes.

These findings have potential clinical implications for HIV-infected persons. The association between increased interferon response and greater odds of depressive symptoms may have relevance for future diagnostic and treatment options [29, 43]. The additive effects of HIV and smoking on inflammatory and stress response transcriptional changes suggests possible mechanisms that may contribute to the increased prevalence and severity of smoking-associated comorbidities among HIV+ individuals [44], and underscores the importance of smoking cessation efforts in this population. Additionally, genes not previously associated with smoking in monocytes identified here may provide new insights into mechanisms specific to HIV+ individuals. In particular, *KLF4* encodes the Krüppel-like factor 4 transcription factor induced by interferon-γ and other pro-inflammatory cytokines in a Stat1-dependent manner in human macrophages and THP-1 monocyte-derived cell lines [45]. Klf4 is involved in diverse biological processes [46], including inflammatory monocyte differentiation [47], and vascular inflammation in both HIV-infected persons and the general population [48, 49]. In this study, *KLF4* was among top DE genes upregulated in HIV+ nonsmokers and further upregulated in HIV+ smokers (Fig. 4). *KLF4* has been associated with tobacco smoking in previous studies of human airway epithelial cells [50] and pulmonary artery smooth muscle cells [51]. Pathway analysis (Fig. 5) identified Klf4 as an indirect positive regulator of APAF1 via caspase, which are involved in inflammation and apoptotic pathways associated with tobacco smoke and HIV infection [44]. Additionally, mitochondria-associated genes were upregulated in tobacco smokers, including *SQOR* (sulfide quinone oxidoreductase, involved in sulfide detoxification), *CISD1* (CDGSH iron sulfur domain protein 1, involved in cellular respiration), and *CYP27A1* (cytochrome P450 enzyme). These findings are consistent with known effects of ROS and oxidative stress on mitochondrial dysfunction in response to tobacco smoke [44, 52].

Limitations of this study include the potential for selection biases in MACS recruitment and the possibility that results observed for MACS participants may not be generalizable to other populations of HIV+ individuals. Tobacco smoking exposures were based on self-report, with limited data on the number of cigarettes smoked or past duration and extent of tobacco use in former smokers, and expression fold-changes for smoking-associated DE genes were low. However, we detected a set of common smoking-associated genes typically differentially expressed with low fold-changes in monocytes reported in previous studies [14–16, 21]. Previous studies collecting detailed smoking exposure data during the same calendar period in large MACS cohorts reported comparable proportions of current smokers among HIV+ participants (20–30%), with roughly twice as many heavy (≥ 1/2 packs/day) compared with light smokers (> 0–< 1/2 packs/day) [7]. Therefore, most tobacco smokers in this study were likely to be heavy smokers. Samples were collected over a four-year interval given the large number of participants, which necessitated processing arrays in batches which could introduce difficult to identify technical artefacts. This concern is mitigated in part by careful exploratory analyses performed here to identify microarrays with QC problems and use of linear models with adjustments for batch and array quality weights. All HIV+ participants were on ART at the time of sample collection, but detailed medication data was available for only 21 participants (38% on NNRTI-based regimens, 48% PI-based regimens, 10% PI + NNRTI-based regimens, 5% INSTI-based regimens); regimens did not differ by tobacco smoking or marijuana use (*p* = 0.557 and *p* = 0.730, respectively; Fisher’s exact test). Our recent study of tobacco and marijuana smokers in a large cohort from the MACS [7] found similar proportions of regimens from 988 participants during the same 2011–2015 calendar period (42% on NNRTI-based regimens, 32% PI-based regimens, 4% PI + NNRTI-based regimens, 20% INSTI-based regimens) and no difference in regimens between tobacco or marijuana smokers (*p* = 0.165 and *p* = 0.577, respectively; Fisher’s exact test). Therefore, ART regimens were likely to be similar in participants with missing data, and that differences in ART medications did not substantially affect gene expression differences by smoking. Monocyte isolation was performed by negative selection (fraction purities ~80% [53]), and therefore the presence of other cell types may have hindered detection of smoking-associated expression differences. Nonetheless, we did not detect substantial expression or variance of other common immune cell type marker genes in exploratory analyses (*CD4*, *CD8*, *CD19*, not shown), and analyses were based on relative comparisons to samples from non-smokers processed with the same methods. The number of participants in this study was low compared with studies of smokers in the general population, and samples were not available to conduct validation experiments with other expression profiling methods such as qRT-PCR. Lastly, there was an insufficient number of samples collected from HIV− smokers (*n =* 7), so we were unable to assess HIV and smoking status as separate terms in models. Given our results and these limitations, future studies are needed with larger cohorts and sensitive expression profiling methods to further evaluate the effects of smoking on monocyte gene expression profiles in people with and without HIV.

## Conclusions

This study describes the effects of tobacco smoking on the transcriptional profiles of monocytes from ART-treated HIV+ and HIV− men with similar demographics and lifestyles. Upregulated genes and enriched GO functional categories reported in previous transcriptomic studies of monocytes from smokers in the general population were observed, while new findings that may be more specific to HIV+ individuals include upregulation of the transcription factor *KLF4* in response to tobacco smoke in monocytes, increased expression of genes related to mitochondria and exosome/extracellular vesicles in HIV+ smokers, and augmented immune/interferon response gene expression in HIV+ persons who also smoke tobacco. Depressive symptoms were more prevalent in HIV+ smokers compared with HIV+ and HIV− non-smokers, and increased expression of immune/interferon response genes was associated with increased odds of depressive symptoms in logistic regression models adjusted for HIV status and smoking. These findings provide a better understanding of immune and stress system responses to tobacco smoking, and identify immune-mediated mechanisms likely to play a role in smoking-related comorbidities in HIV+ individuals.

## Methods

### Study population

Individuals enrolled in the MACS are seen at biannual study visits at which they donate biological specimens, undergo physical examinations, and complete standardized interviews detailing substance use, behavioral characteristics, and medical conditions or treatments. Participants for this study were MACS subjects at the University of Los Angeles, California (UCLA) recruited for a previous sub-study investigating peripheral blood monocyte gene expression biomarkers associated with neurocognitive impairment [53]. Blood draws were performed between 2011 and 2015 according to procedures previously described [53, 54]. All participants were on ART at the time of sample collection.

### Blood processing, monocyte isolation, RNA extraction, and gene expression profiling

Details of sample collection and gene expression profiling have been previously described [53, 54]. Briefly (Fig. 1), 24 ml of fresh blood was collected from each participant into three 8 ml cell preparation tubes containing sodium citrate. Peripheral blood mononuclear cells (PBMCs) were isolated by centrifugation within 6 hours of collection, washed with phosphate-buffered saline, and monocytes were isolated by negative selection using RosetteSep™ separation per manufacturer’s instructions (Stem Cell Technologies, Vancouver, BC). RNA was extracted from isolated monocytes using the QIAGEN RNeasy kit, which included DNase treatment, according to manufacturer’s instructions (QIAGEN Inc., Germantown, MD). Microarray analyses were performed at the Southern California Genotyping Center (SCGC), which included Agilent Bioanalyzer quality control (QC) (Agilent Technologies, Santa Clara, CA), biotinylated cRNA preparation using the Ambion MessageAmp kit for Illumina arrays (Thermo Fisher Scientific, Waltham, MA), and gene expression profiling using the Illumina Human HT-12 v4 gene expression BeadChip arrays (Illumina Inc., San Diego, CA). Expression and sample data in MIAME standard format were deposited to the NCBI GEO database (accession no. GSE104640), from which quantile-normalized and raw probe intensity and detection data for this study were obtained.

### Sample covariates

Tobacco smoking was the primary exposure of interest. Participants reported if they were current tobacco smokers (TS+), or former or never smokers (TS−) at the time of the study visit. Marijuana, cocaine, and alcohol use were defined as daily or weekly use of those substances during the prior six months. Age, race (white, black, Hispanic, or other race/ethnicity), and years of education were self-reported. Participants with a total score of 16 or greater on the Center for Epidemiologic Studies Depression Scale (CES-D) [55] were classified as having depressive symptoms. Additional covariates examined included HIV serostatus, body mass index (BMI), hepatitis C serostatus, HIV viral load, and CD4+ T cell count, which were measured as previously described [53].

### Microarray data preprocessing and quality control

An overview of the data processing workflow is included in Fig. 1. Samples were selected from the 248 microarrays in the GEO data set to exclude outlier arrays with QC issues while retaining sufficient numbers of TS+ samples, and balanced proportions of samples from TS+ vs. TS− participants. Outlier samples were identified from quantile-normalized, log_2_-transformed data by sample distance hierarchical clustering, principal component analysis (PCA), and exploratory plots of selected immune cell type-specific and housekeeping genes. One hundred twenty-five samples were selected including 40 samples from HIV− TS−, 60 from HIV+ TS−, and 25 from HIV+ TS+ participants; there were insufficient numbers of arrays sampling HIV− TS+ participants (*n* = 4) for inclusion.

The *illuminaHumanv4.db_v1.26.0* R/Bioconductor package was used to reannotate or omit 13,631 of 48,107 total probes with “Bad” and “No match” hybridization specificity (mappings based on data from NCBI Entrez Gene, March 17, 2015). A total of 5706 probes with missing or discrepant gene mappings were verified or re-assigned by sequence similarity search using remote *blastn*_*v2.9.0* (data from NCBI Entrez Gene, April 25, 2019); probes with ≥ 95% identity and ≥ 95% length match to a unique feature were retained. Probes mapping to putative/hypothetical/antisense ORFs or pseudogenes, and probes detected below background (detection *p* value ≥ 0.05) in all samples were excluded from downstream analyses. Normalization and generalized logarithm, base 2 (glog_2_) transformation of the remaining 24,941 raw probe intensities was performed using the *vsn_v3.50.0* (variance stabilizing normalization) R/Bioconductor package.

### Differential expression analysis

Sample PCA and distance clustering of *vsn*-normalized probe data was repeated, which identified samples from three HIV+ TS+ and three HIV+ TS− participants as technical outliers that were excluded from subsequent analyses (Additional file 1, Figure S5). Filtering was performed to retain probes expressed above background in 22 or more samples (size of the smallest exposure group) and remove low-variance probes in the lowest interquartile range tertile. Differential expression (DE) analysis of the 10,534 remaining probes was performed using the R/Bioconductor *limma_v3.36.5* package with recommended adjustments for batch and estimated relative array quality weights, and control of multiple testing as previously described [56]. Models were fit contrasting: (A) 22 HIV+ TS+ vs. 40 HIV− TS−, and (B) 22 HIV+ TS+ vs. 47 HIV+ TS−, and (C) 47 HIV+ TS− vs. 40 HIV− TS−. Ten HIV+ TS− participants who reported daily or weekly marijuana use, most likely by smoking, were excluded from the HIV+ TS− group. Fold-changes for each probe were estimated following empirical Bayes moderation of standard errors; *p* values were adjusted for false discovery rate (FDR) using the Benjamini-Hochberg method. To identify sufficient numbers of DE genes from models B and C for comparisons with model A and downstream functional enrichment analysis, lower stringency cutoffs (log_2_ fold-change (FC) > 0.25, FDR-adjusted *p* value < 0.15) were used to identify DE probes from all models.

### Gene ontology and functional analysis

Functional category enrichment of gene ontology (GO) terms was performed using the R/Bioconductor *goana* and *topGO* functions in the *limma_v3.36.5* package. GO term-to-gene mapping was obtained from the *org.Hs.eg.db_v3.6.0* package (October, 2018). Enrichment analysis was performed using Fisher’s exact test with 7945 distinct genes mapped to the 10,534 expressed probes as the reference set. Genes with an absolute log_2_ fold-change > 0.25 and FDR-adjusted *p* value < 0.15 were included in GO term enrichment analysis, which was performed separately for up- and down-regulated probes. Terms with ≥ 5 associated genes and ≥ 2 differentially expressed genes in each experiment were included; FDR adjustment of *p* values was performed using the Benjamini-Hochberg method.

### Additional statistical analyses

All data preparation and analyses were performed in R version 3.5.1. Demographic and clinical covariates were compared using Fisher’s exact test or Kruskal-Wallis test for categorical and continuous variables, respectively. Data were visualized using the R *ComplexHeatmap_v1.18.1* package, *ggpubr_v0.2* with Wilcoxon rank-sum tests for box/dot plots, and *ggplot2*_*v3.1.0*. *K*-means clustering analyses of selected gene subsets were performed with *z-*scored glog_2_ probe intensity data using the R *kmeans* function; optimal values for *k* centers were estimated using the elbow method comparing within-cluster sum of squares errors vs. *k*. Logistic regression models were used to assess the association between immune and interferon-associated gene expression and CES-D ≥ 16. Models were fit using a continuous variable (normalized glog_2_ probe intensities) and a binary categorical variable (highest vs. combined middle and lowest expression tertiles) for each gene; all models were adjusted by HIV status and smoking. Network analysis was performed using Ingenuity Pathway Analysis software (QIAGEN Inc., Germantown, PA). Pearson correlation analyses between the set of 10,534 filtered probes and known smoking-associated *SASH1* gene (probe ID ILMN_2185984) were performed with the R *cor.test* function; *p* values were FDR-adjusted using the tail-area based method from the *fdrtool_v1.2.15* package.

### Sensitivity analyses

To assess the effect of including HIV+ participants with unsuppressed HIV viral load, arrays from two TS+ and four TS− participants with viral load > 200 copies/ml at time of sample collection were omitted, and differential expression and gene ontology enrichment analyses were repeated.

## Supplementary information

**Additional file 1: Figure S1.** Overview and comparison of differentially expressed gene sets. (**a**) Number of differentially expressed genes from models contrasting HIV+ smokers vs. HIV- and HIV+ non-smokers and Venn diagrams of overlapping up- and down-regulated gene sets; (**b**) Number of differentially expressed genes from models contrasting HIV+ smokers vs. HIV- and HIV+ non-smokers in sensitivity analyses excluding HIV+ participants with HIV viral load > 200 and Venn diagrams of overlapping up- and down-regulated gene sets; (**c**) Venn diagrams comparing sets of up- and down-regulated genes in full cohort analyses vs. HIV viral load > 200 excluded cohort, with arrays from HIV- non-smokers as the reference; and (**d**) Venn diagrams comparing sets of up- and down-regulated genes in full cohort analyses vs. HIV viral load > 200 excluded cohort, with HIV+ nonsmokers as the reference. Genes with FDR-adjusted *p* value < 0.15, absolute log_2_ FC > 0.25 were deemed differentially expressed for all comparisons. **Figure S2.** Expression levels of genes associated with increased depressive symptoms stratified by HIV status, tobacco smoking, and CES-D score ≥ 16. Boxplots of normalized expression levels for genes associated with CES-D ≥ 16 in multivariate logistic regression models (Table 2). Values from five HIV- participants with CES-D ≥ 16 were excluded from plots for clarity. Horizontal bars denote medians, boxes span IQRs, whiskers extend to 1.5 x IQR. Dots denote expression values from individual arrays. Groups were compared using the Wilcoxon rank-sum test. Abbreviations: CES-D, Center for Epidemiological Studies Depression Scale; CES-D–, CES-D score < 16; CES-D16+, CES-D score ≥ 16; IQR, interquartile range; TS+, tobacco smoker; TS-, non-smoker. **Figure S3.** Expression levels of selected immune/interferon response, stress/chemical response, mitochondrion, and smoking-associated genes stratified by HIV status and marijuana or tobacco smoking. Boxplots of normalized gene expression levels for selected genes in Figure 4. Horizontal bars denote medians, boxes span IQRs, whiskers extend to 1.5 x IQR. Dots denote expression values from individual arrays. Groups were compared using the Wilcoxon rank-sum test. Abbreviations: IQR, interquartile range; MJ+, marijuana smoker; MJ-, marijuana non-smoker; TS+, tobacco smoker; TS-, non-smoker. **Figure S4.** Genes with high correlation to known smoking-associated gene *SASH1* in HIV+ smokers and HIV- non-smokers. Correlation analyses of eight DE genes in HIV+ smokers vs. HIV- non-smokers showing significant correlation with *SASH1* expression. Probe intensities were normalized and generalized-log_2_ transformed using the R/Bioconductor *vsn* package. Dots denote probe intensities from individual arrays, r denotes Pearson’s correlation coefficient, q denotes FDR-adjusted *p* value. Abbreviations: FDR, false-discovery rate; TS+, tobacco smoker; TS-, non-smoker. **Figure S5**. Identification of outlier samples by principal component analysis (PCA). Scatter plot of the first two principal components calculated from the set of 24,941 normalized probe intensities (*n* = 125 microarrays). Outlier samples removed from subsequent differential expression analyses are marked as red triangles (*n* = 6). Abbreviations: PC, principal component

**Additional file 2: Table S1.** Fold-changes for all expressed probes: HIV+ TS+ vs. HIV- TS- *limma* model A. **Table S2.** Enriched gene ontology (GO) terms and associated genes: HIV+ TS+ vs. HIV- TS-. **Table S3.** Fold-changes for all expressed probes: HIV+ TS+ vs. HIV+ TS- *limma* model B. **Table S4.** Enriched gene ontology (GO) terms and associated genes: HIV+ TS+ vs. HIV+ TS-. **Table S5.** Fold-changes for all expressed probes: HIV+ TS- vs. HIV+ TS- *limma* model C. **Table S6.** Enriched gene ontology (GO) terms and associated genes: HIV+ TS- vs. HIV+ TS-. **Table S7.** Fold-changes for all expressed probes: HIV+ (HIV viral load ≤200) TS+ vs. HIV- TS- *limma* model for sensitivity analysis. **Table S8.** Enriched gene ontology (GO) terms and associated genes: HIV+ (HIV viral load ≤200) TS+ vs. HIV- TS-. **Table S9.** Fold-changes for all expressed probes: HIV+ (HIV viral load ≤200) TS+ vs. HIV+ (HIV viral load ≤200) TS- *limma* model for sensitivity analysis. **Table S10.** Enriched gene ontology (GO) terms and associated genes: HIV+ (HIV viral load ≤200) TS+ vs. HIV+ (HIV viral load ≤200) TS-.

## Data Availability

The data used for the current study are available from the NCBI Gene Expression Omnibus (accession no. GSE104640) at https://www.ncbi.nlm.nih.gov/geo/. We thank Andrew Levine (UCLA) for helpful discussions and depositing the GEO datasets used for this study.
