# Supplementary material for: Transcriptomic analysis of monocytes from HIV-positive men on antiretroviral therapy reveals effects of tobacco smoking on interferon and stress response systems associated with depressive symptoms
Source: Hum Genomics. 2019 Nov 28;13:59. doi: 10.1186/s40246-019-0247-x (PMC6883692; doi:10.1186/s40246-019-0247-x)
Supplement: Supplementary file 1 — Additional file 1: Figure S1. Overview and comparison of differentially expressed gene sets. (a) Number of differentially expressed genes from models contrasting HIV+ smokers vs. HIV- and HIV+ non-smokers and Venn diagrams of overlapping up- and down-regulated gene sets; (b) Number of differentially expressed genes from models contrasting HIV+ smokers vs. HIV- and HIV+ non-smokers in sensitivity analyses excluding HIV+ participants with HIV viral load > 200 and Venn diagrams of overlapping up- and down-regulated gene sets; (c) Venn diagrams comparing sets of up- and down-regulated genes in full cohort analyses vs. HIV viral load > 200 excluded cohort, with arrays from HIV- non-smokers as the reference; and (d) Venn diagrams comparing sets of up- and down-regulated genes in full cohort analyses vs. HIV viral load > 200 excluded cohort, with HIV+ nonsmokers as the reference. Genes with FDR-adjusted p value < 0.15, absolute log2 FC > 0.25 were deemed differentially expressed for all comparisons. Figure S2. Expression levels of genes associated with increased depressive symptoms stratified by HIV status, tobacco smoking, and CES-D score ≥ 16. Boxplots of normalized expression levels for genes associated with CES-D ≥ 16 in multivariate logistic regression models (Table 2). Values from five HIV- participants with CES-D ≥ 16 were excluded from plots for clarity. Horizontal bars denote medians, boxes span IQRs, whiskers extend to 1.5 x IQR. Dots denote expression values from individual arrays. Groups were compared using the Wilcoxon rank-sum test. Abbreviations: CES-D, Center for Epidemiological Studies Depression Scale; CES-D–, CES-D score < 16; CES-D16+, CES-D score ≥ 16; IQR, interquartile range; TS+, tobacco smoker; TS-, non-smoker. Figure S3. Expression levels of selected immune/interferon response, stress/chemical response, mitochondrion, and smoking-associated genes stratified by HIV status and marijuana or tobacco smoking. Boxplots of normalized gene expression leve [file 40246_2019_247_MOESM1_ESM.pdf]

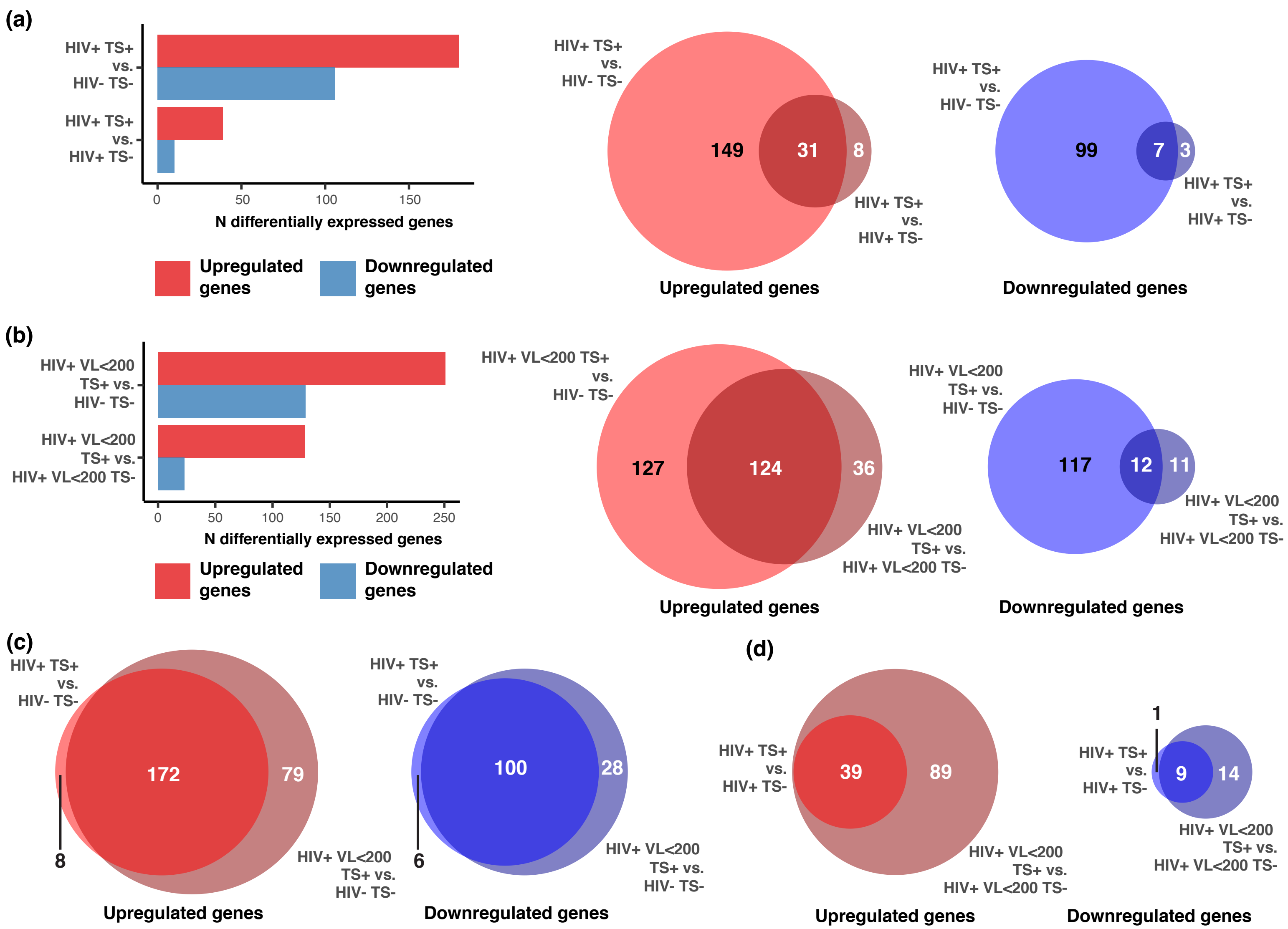

Figure S1. **Overview and comparison of differentially expressed gene sets. (a)** Number of differentially expressed genes from models contrasting HIV+ smokers vs. HIV- and HIV+ non-smokers and Venn diagrams of overlapping up- and down-regulated gene sets; **(b)** Number of differentially expressed genes from models contrasting HIV+ smokers vs. HIV- and HIV+ non-smokers in sensitivity analyses excluding HIV+ participants with HIV viral load >200 and Venn diagrams of overlapping up- and down-regulated gene sets; **(c)** Venn diagrams comparing sets of up- and down-regulated genes in full cohort analyses vs. HIV viral load >200 excluded cohort, with arrays from HIV- non-smokers as the reference; and **(d)** Venn diagrams comparing sets of up- and down-regulated genes in full cohort analyses vs. HIV viral load >200 excluded cohort, with HIV+ nonsmokers as the reference. Genes with FDR-adjusted *p* value <0.15, absolute $\log_2$ FC >0.25 were deemed differentially expressed for all comparisons.

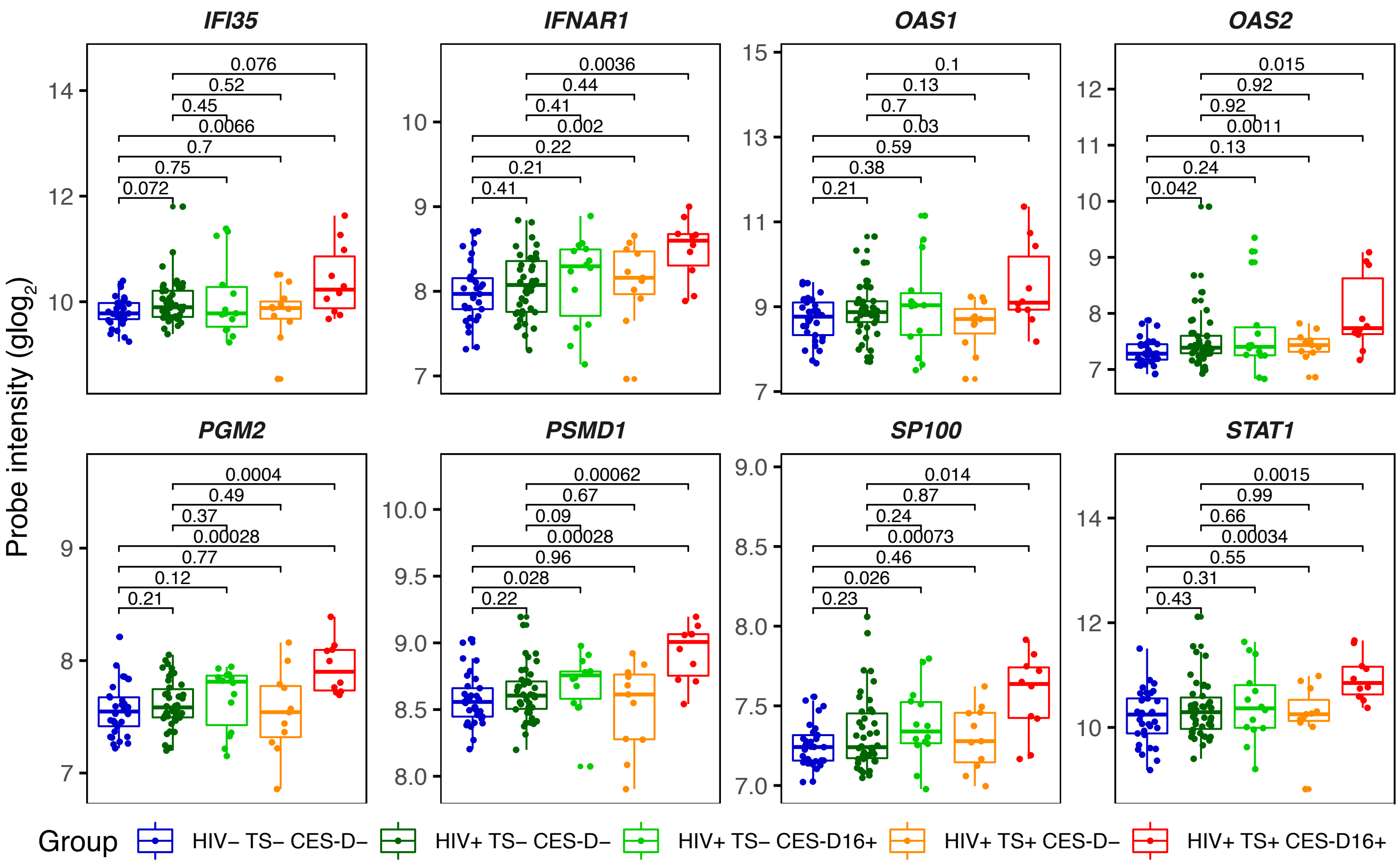

Figure S2. Expression levels of genes associated with increased depressive symptoms stratified by HIV status, tobacco smoking, and CES-D score ≥16. Boxplots of normalized expression levels for genes associated with CES-D ≥16 in multivariate logistic regression models (Table 2). Values from five HIV- participants with CES-D ≥16 were excluded from plots for clarity. Horizontal bars denote medians, boxes span IQRs, whiskers extend to 1.5 x IQR. Dots denote expression values from individual arrays. Groups were compared using the Wilcoxon rank-sum test. Abbreviations: CES-D, Center for Epidemiological Studies Depression Scale; CES-D–, CES-D score <16; CES-D16+, CES-D score ≥16; IQR, interquartile range; TS+, tobacco smoker; TS-, non-smoker.

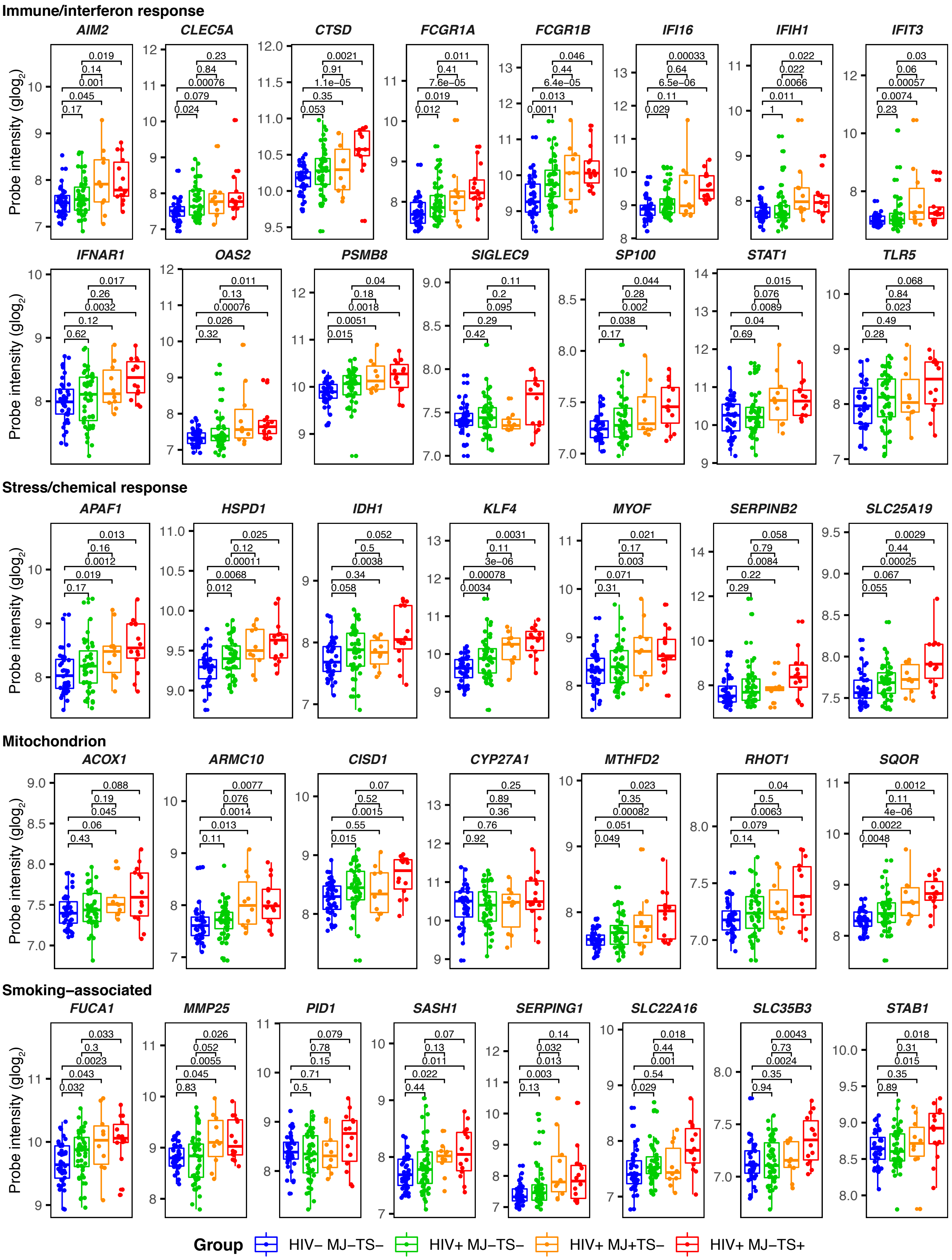

Figure S3. Expression levels of selected immune/interferon response, stress/chemical response, mitochondrion, and smoking-associated genes stratified by HIV status and marijuana or tobacco smoking. Boxplots of normalized gene expression levels for selected genes in Figure 4. Horizontal bars denote medians, boxes span IQRs, whiskers extend to 1.5 x IQR. Dots denote expression values from individual arrays. Groups were compared using the Wilcoxon rank-sum test. Abbreviations: IQR, interquartile range; MJ+, marijuana smoker; MJ-, marijuana non-smoker; TS+, tobacco smoker; TS-, non-smoker.

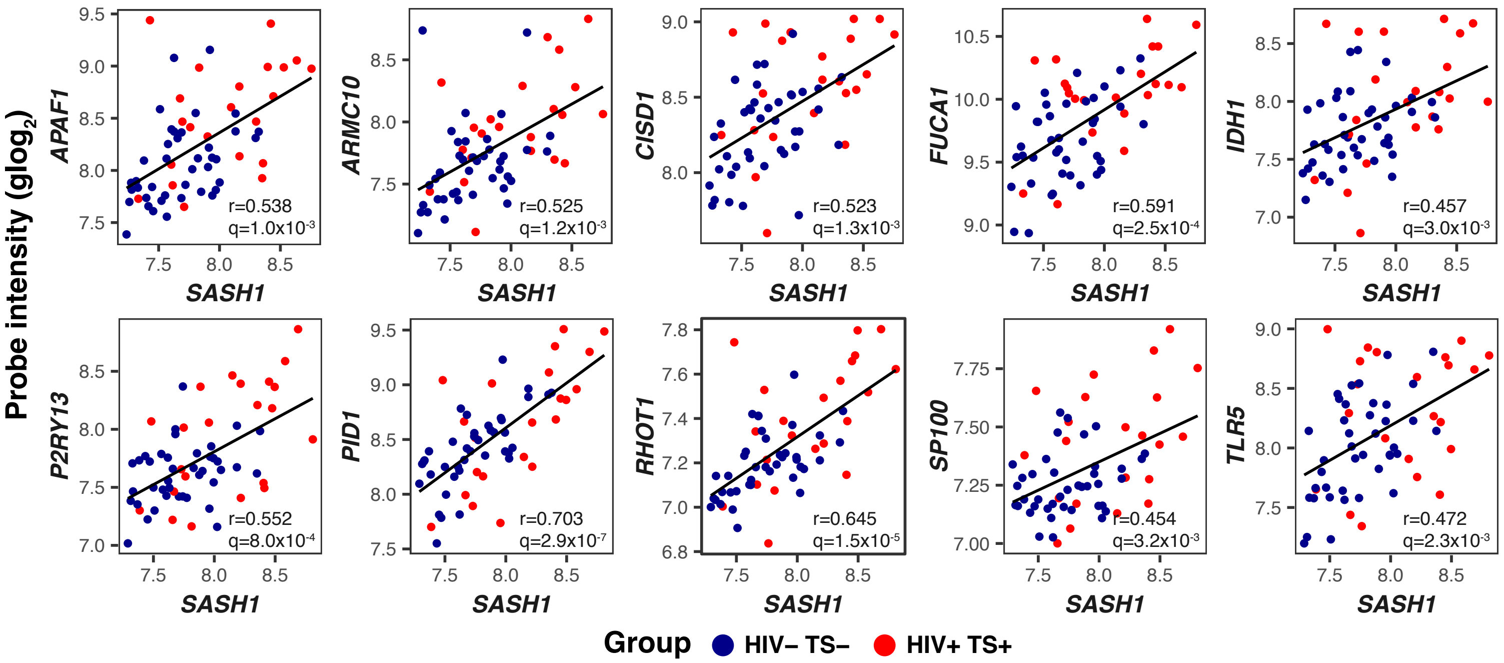

Figure S4. Genes with high correlation to known smoking-associated gene *SASH1* in HIV+ smokers and HIV- non-smokers. Correlation analyses of eight DE genes in HIV+ smokers vs. HIV- non-smokers showing significant correlation with *SASH1* expression. Probe intensities were normalized and generalized-log_2 transformed using the R/Bioconductor *vsn* package. Dots denote probe intensities from individual arrays, *r* denotes Pearson's correlation coefficient, *q* denotes FDR-adjusted *p* value. Abbreviations: FDR, false-discovery rate; TS+, tobacco smoker; TS-, non-smoker.

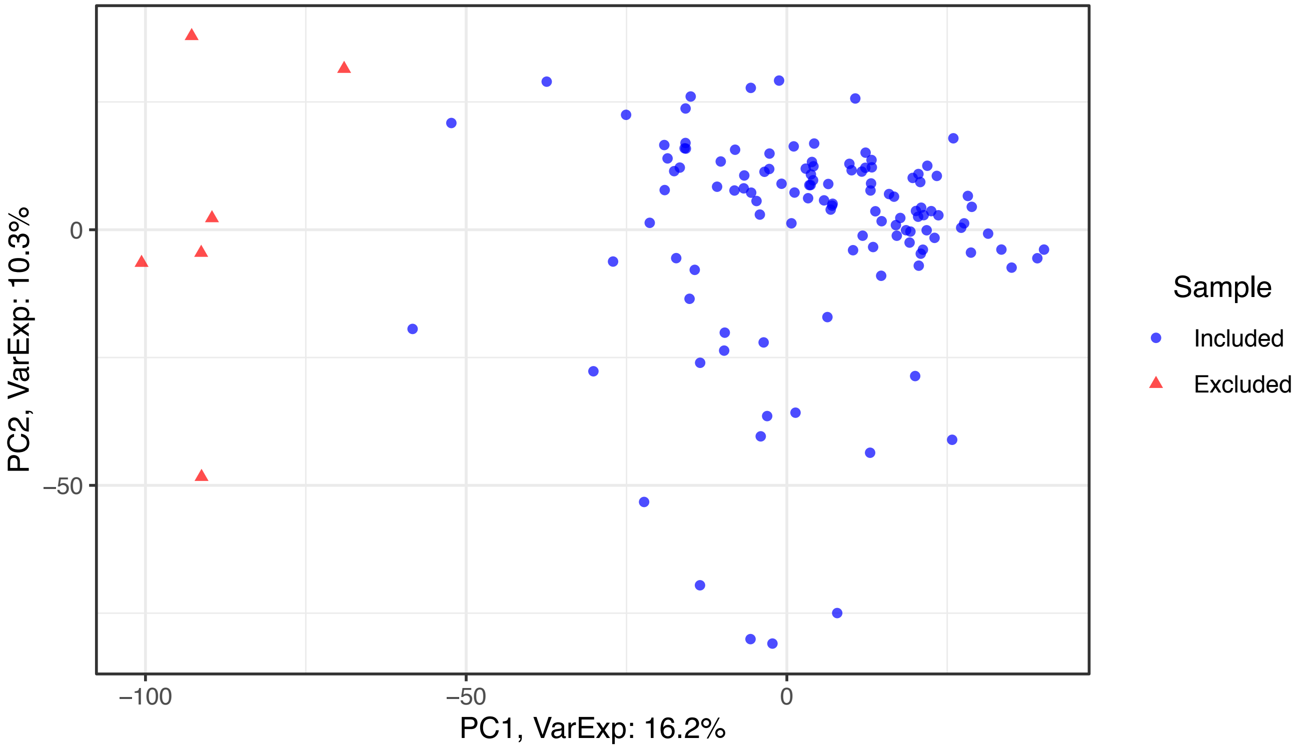

Figure S5. Identification of outlier samples by principal component analysis (PCA). Scatter plot of the first two principal components calculated from the set of 24,941 normalized probe intensities (n=125 microarrays). Outlier samples removed from subsequent differential expression analyses are marked as red triangles (n=6). Abbreviations: PC, principal component.
